# The effect of hyperbaric oxygen therapy on sleep quality across diverse patient populations

**DOI:** 10.3389/fneur.2026.1690633

**Published:** 2026-03-30

**Authors:** Keren Doenyas-Barak, Karin Elman Shina, Erez Lang, Shachar Finci, Vicktoria Elkarif, Ran Shorer, Shai Efrati

**Affiliations:** 1 Yitzhak Shamir Medical Center, Tel Aviv, Israel; 2 Faculty of Medical and Health Sciences, Gray School of Medicine, Tel Aviv University, Tel Aviv, Israel

**Keywords:** aging, COVID-19, hyperbaric oxygen (HBO) therapy, neuroplasticity, Pittsburgh Sleep Quality Index (PSQI), PTSD, sleep

## Abstract

**Background:**

Sleep disturbances are common in aging, post-traumatic stress disorder (PTSD), and long COVID, often linked to neuroinflammation, autonomic dysregulation, and neurodegeneration. Hyperbaric oxygen therapy (HBOT) promotes neuroplasticity through the hyperoxic-hypoxic paradox, improving cerebral perfusion, mitochondrial function, and reducing inflammation. While HBOT benefits sleep in certain conditions, its general effects across clinical populations remain unclear.

**Methods:**

This retrospective longitudinal study evaluated Pittsburgh Sleep Quality Index (PSQI) changes in patients undergoing 60 HBOT sessions (2.0 ATA, 100% oxygen, 90 min, 5 days/week) at the Sagol Center. Participants included individuals treated for healthy aging (*n* = 180), long COVID (*n* = 92), or PTSD (*n* = 123). Pre- and post-treatment PSQI total and component scores were compared using paired t-tests and Wilcoxon signed-rank test was used for PSQI components. Regression analysis identified predictors of improvement.

**Results:**

Among 395 patients (mean age at baseline 57.9 ± 14.6 years, 31% female), baseline PSQI scores were highest in PTSD. Post-HBOT, total PSQI scores improved significantly in all groups (*p* < 0.001; Cohen’s d = 0.37–0.91). Significant gains were observed in subjective sleep quality, sleep latency, and disturbances in all groups; daytime dysfunction improved in aging and long COVID but not PTSD. Medication use was unchanged. Baseline PSQI was a strong predictor of improvement (B = 0.494, *p* < 0.001, r = 0.46). Those with disturbed sleep (PSQI>5) showed broad, statistically significant gains, while normal sleepers exhibited minimal changes.

**Conclusion:**

HBOT was associated with improvement in sleep quality across diverse conditions, with greatest benefit in patients with poorer baseline sleep. Findings support HBOT’s potential as a sleep-modulating therapy, warranting controlled trials to characterize the patients that can benefit the most and elucidate mechanisms.

## Background

Sleep is a fundamental physiological process characterized by altered consciousness, reduced responsiveness to external stimuli, and restorative neural activity. Adequate sleep is crucial for maintaining both physiological and psychological functioning, especially regarding brain performance and homeostasis (1, 2). Poor sleep can contribute to, and result from, impaired brain function (3).

Sleep disturbances, including fragmented sleep, REM sleep behavior disorder, and excessive daytime sleepiness, are common in neurodegenerative diseases such as Parkinson’s disease and are driven by underlying neurodegenerative changes (4, 5). Sleep disturbances are also prevalent in conditions like PTSD (6) and Long COVID (7), where they are associated with underlying neuroinflammation (8) and dysregulated autonomic function.

Sleep quality naturally declines with age (9), marked by shorter sleep duration, increased fragmentation, reduced slow-wave sleep, and alterations in circadian rhythms and hormone secretion patterns. Impaired sleep in older adults is associated with diminished cognitive function (10), increased cardiovascular and metabolic risk (11, 12), weakened immune responses, and heightened susceptibility to chronic illnesses.

Hyperbaric oxygen therapy (HBOT), involving the inhalation of pure oxygen at supra-atmospheric pressures, has emerged as a neuromodulatory intervention. Dedicated HBOT protocols, employing the hyperoxic-hypoxic paradox (HHP), through cyclic oxygen fluctuations, activate neurobiological pathways including hypoxia-inducible factors (HIF), vascular endothelial growth factor (VEGF), stem cell proliferation, angiogenesis, and mitochondrial biogenesis, alongside reductions in neuroinflammation (13–15). These mechanisms underlie the neuroplasticity and clinical improvements observed in stroke, traumatic brain injury, post-COVID syndrome, PTSD, and age-related functional decline (14, 16–23).

Several studies suggest potential beneficial effects of HBOT on sleep quality across distinct clinical populations (24–26). For example, HBOT has been associated with improved sleep duration and reduced sleep latency in children with cerebral palsy (25). In Parkinson’s disease, a meta analysis and a systematic review reports association with enhanced sleep efficiency, increased total sleep duration, reduced sleep latency, and fewer awakenings following HBOT (27, 28). Similarly, patients with PTSD experience reduced nightmares and improved sleep quality following HBOT (29).

Although emerging evidence suggests that HBOT may influence sleep-related outcomes in specific clinical contexts, existing studies are limited by small sample sizes, heterogeneous populations, and varying outcome measures. This retrospective study aimed to assess HBOT’s impact on sleep quality among patients treated for various clinical indications by using subjective reports in sleep quality questionnaires.

## Methods

The study is a retrospective, longitudinal analysis evaluating changes in sleep quality, assessed by the Pittsburgh Sleep Quality Index (PSQI), among patients completing a 60-session HBOT protocol at the Sagol Center for Hyperbaric Medicine and Research.

### Study population

The study followed the STROBE (Strengthening the Reporting of Observational Studies in Epidemiology) reporting recommendations. Patients were identified from the clinical database of the Sagol Center for Hyperbaric Medicine and Research. All adult patients who completed a 60-session hyperbaric oxygen therapy (HBOT) protocol during the study period and had available PSQI assessments at baseline and after completion of the treatment course were screened for eligibility.

A total of 409 patients completed the HBOT protocol and had available PSQI data. Among these, 14 patients were excluded because the PSQI questionnaires were not completed properly, preventing calculation of the global PSQI score. Consequently, the final analytic cohort included 395 patients with complete PSQI assessments at both time points. The final cohort consisted of patients treated for healthy aging (*n* = 180), long COVID (*n* = 92), and post-traumatic stress disorder (PTSD) (*n* = 123). The healthy aging group included individuals aged 50 years or older without diagnosed neurodegenerative disease or pathological cognitive decline, who were living independently and had preserved functional and cognitive status. These patients recieved HBOT as part of a program aimed at improving age-related functional decline and cognitive performance. The long COVID group included patients reporting persistent cognitive symptoms following SARS-CoV-2 infection that affected their quality of life and persisted for more than three months after confirmed symptomatic COVID-19 infection, consistent with commonly used definitions of post-COVID condition. The PTSD group consisted of patients with a clinical diagnosis of post-traumatic stress disorder established by either the Israeli Ministry of Defense or a psychiatrist from a health maintenance organization, with persistent debilitating symptoms despite prior psychological and pharmacological treatment.

**Figure 1:**
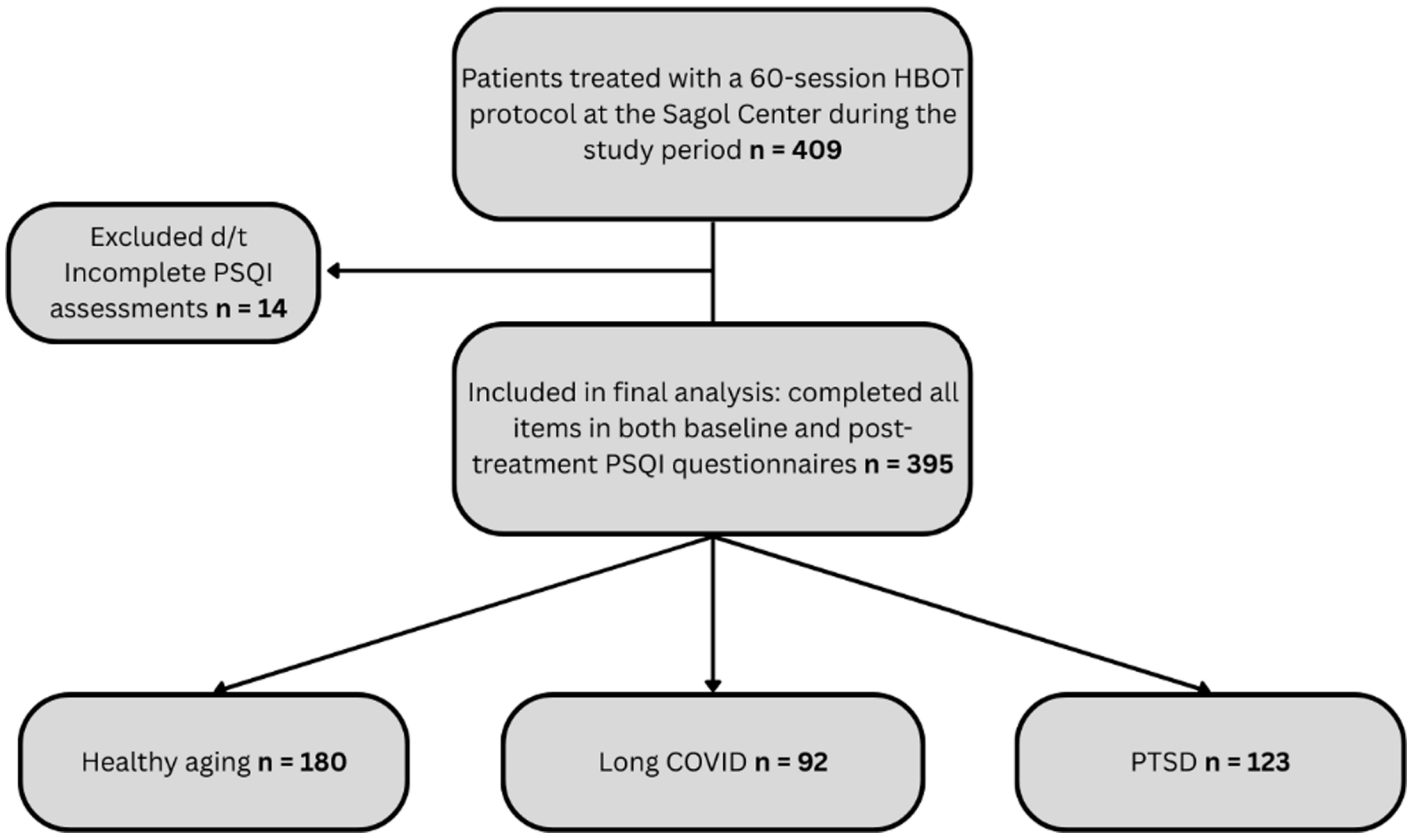
STROBE flow diagram of participant selection.

A flow diagram illustrating participant selection and exclusion is presented in Figure 1. Detailed demographic and clinical characteristics of the study population, including sample size, age, and sex distribution, are presented in the Results section and Table 1.

**Table 1:** Patients baseline characteristics.

	All patients	Aging	Long COVID	PTSD
*N*	395	180	92	123
Age	57.9 ± 14.6	65.8 ± 9.8	51.23 ± 13.4	51.4 ± 15.6
Female (%)	138 (32)	65 (36)	39 (42)	22 (18)
HTN	51 (13)	34 (19)	14 (15)	3 (2)
DM	4 (1)	2 (1)	1 (1)	1 (1)
IHD	26 (7)	17 (9)	6 (7)	3 (2)
Heart disease	31 (8)	20 (11)	5 (5)	6 (5)
CVD	25 (6)	17 (9)	4 (4)	4 (3)
OSA	10 (3)	4 (2)	3 (3)	3 (2)
BPH	3 (1)	3 (2)	0 (0)	0 (0)
SSRI/SNRI	67 (17)	13 (7)	6 (7)	48 (39)
BDZ	36 (9)	1 (1)	2 (2)	33 (27)
BB	29 (7)	12 (7)	4 (4)	11 (9)
Alpha block	24 (6)	12 (7)	2 (2)	10 (8)

HTN, hypertension; DM, diabetes mellitus; IHD, ischemic heart disease; CVD, cerebrovascular disease; OSA, obstructive sleep apnea; BPH, benign prostatic hypertrophy; SSRI/SNRI, Selective Serotonin Reuptake Inhibitor/Serotonin–Norepinephrine Reuptake Inhibitor BDZ, benzodiazepines; BB, beta blockers.

### HBOT protocol

**Figure 2:**
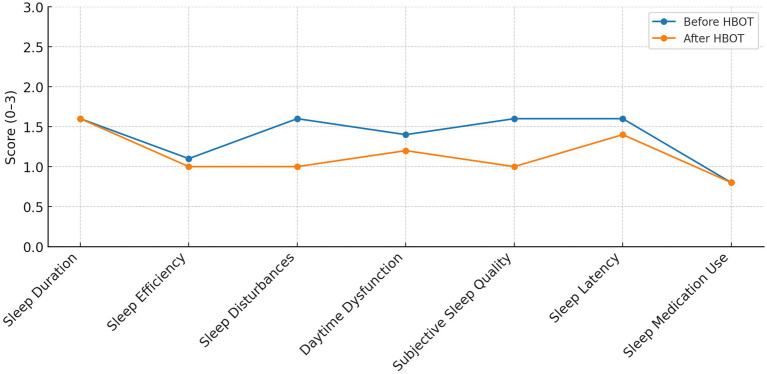
Mean Pittsburgh Sleep Quality Index component scores before and after hyperbaric oxygen therapy.

Patients received 60 daily sessions (five days per week), each lasting 90 min at 2.0 atmospheres absolute (ATA), breathing 100% oxygen with periodic air breaks. Sessions occurred in a multiplace hyperbaric chamber supervised by certified medical staff (Figure 2).

### Assessment

**Figure 3:**
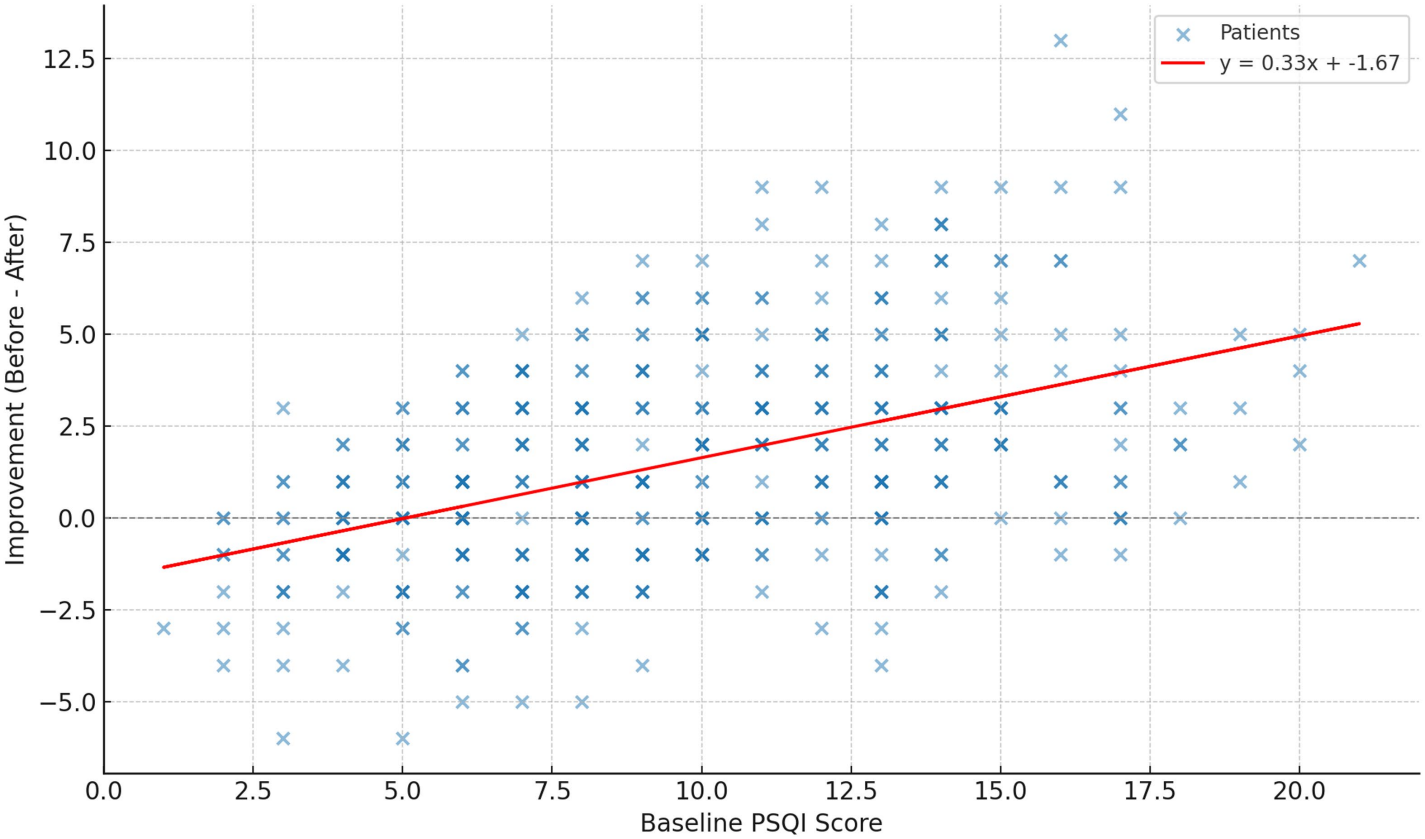
Baseline Pittsburgh Sleep Quality Index score vs. improvement after hyperbaric oxygen therapy. PSQI: Pittsburgh Sleep Quality Index.

Sleep quality was evaluated using the Pittsburgh Sleep Quality Index (PSQI), a widely used and validated self-report questionnaire that assesses sleep patterns and disturbances over the preceding month. The PSQI yields a global score ranging from 0 to 21, with higher scores indicating poorer sleep quality. It consists of seven components including subjective sleep quality, sleep latency, sleep duration, habitual sleep efficiency, sleep disturbances, use of sleep medications, and daytime dysfunction, each scored on a 0–3 scale, with the sum representing overall sleep quality (Figure 3).

The PSQI has demonstrated high internal consistency, strong test–retest reliability, and good construct validity across both clinical and general populations. It is sensitive to detecting changes in sleep quality and has been validated in diverse patient groups, including those with neuropsychiatric, neurological, and chronic medical conditions (30, 31) (Table 2).

**Table 2:** HBOT sleep outcomes.

Group	Domain	Pre-HBOT (mean ± SD)	Post-HBOT (mean ± SD)	*p*-value	Cohen’s d
All patients	Total	9.0 ± 4.0	8.2 ± 3.8	<0.001*	0.91
All patients	Duration	1.6 ± 0.92	1.6 ± 0.9	0.029	
All patients	Efficacy	1.1 ± 1.3	1.0 ± 1.24	0.73	
All patients	Sleep Disturbances	1.6 ± 0.66	1.0 ± 0.36	<0.001*	
All patients	Daytime Dysfunction	1.4 ± 0.93	1.2 ± 0.84	<0.001*	
All patients	Subjective Sleep Quality	1.6 ± 0.9	1.0 ± 0.9	<0.001*	
All patients	Latency	1.6 ± 1.0	1.4 ± 0.96	<0.001*	
All patients	Medications	0.8 ± 1.26	0.8 ± 1.23	0.244	
Aging	Total	7.1 ± 3.3	6.5 ± 3.0	<0.001*	0.37
Aging	Duration	1.4 ± 0.81	1.4 ± 0.78	0.511	
Aging	Efficacy	1.0 ± 1.3	0.9 ± 1.2	<0.707	
Aging	Sleep Disturbances	1.4 ± 0.57	1.0 ± 0.21	0.001*	
Aging	Daytime Dysfunction	1.0 ± 0.81	0.8 ± 0.65	<0.001*	
Aging	Subjective Sleep Quality	1.1 ± 0.79	0.9 ± 0.73	<0.001*	
Aging	Latency	1.3 ± 0.89	1.1 ± 0.92	0.019	
Aging	Medications	0.4 ± 0.94	0.4 ± 0.94	0.658	
Long COVID	Total	8.4 ± 3.6	7.7 ± 3.3	<0.001*	0.54
Long COVID	Duration	1.4 ± 0.9	1.2 ± 0.85	0.021	
Long COVID	Efficacy	0.8 ± 1.2	1.1 ± 1.29	0.08	
Long COVID	Sleep Disturbances	1.5 ± 0.64	1.0 ± 0.35	<0.001*	
Long COVID	Daytime Dysfunction	1.7 ± 0.94	1.1 ± 0.74	<0.001*	
Long COVID	Subjective Sleep Quality	1.6 ± 0.89	1.1 ± 0.88	<0.001*	
Long COVID	Latency	1.4 ± 1.0	1.1 ± 0.88	0.002*	
Long COVID	Medications	0.5 ± 1.02	0.48 ± 1.05	0.929	
PTSD	Total	12.1 ± 3.5	10.9 ± 3.6	<0.001*	0.74
PTSD	Duration	2.2 ± 0.89	2.1 ± 0.89	0.3	
PTSD	Efficacy	1.3 ± 1.3	1.0 ± 1.2	0.031	
PTSD	Sleep Disturbances	2.0 ± 0.62	1.1 ± 0.51	<0.001*	
PTSD	Daytime Dysfunction	1.8 ± 0.82	1.7 ± 0.86	0.171	
PTSD	Subjective Sleep Quality	2.2 ± 0.77	1.8 ± 0.83	<0.001*	
PTSD	Latency	2.3 ± 0.86	2.0 ± 0.87	<0.001*	
PTSD	Medications	1.7 ± 1.38	1.6 ± 1.36	0.119	

*Significant after Bonferroni correction for multiple comparisons (α = 0.007). Effect sizes reported only for global PSQI, calculated from paired differences.

### Statistical analysis

Statistical analysis was conducted using SPSS v29.0. Continuous variables were assessed for normality using the Shapiro–Wilk test. Normally distributed paired data (e.g., total PSQI score) were analyzed using the paired Student’s t-test. Because individual PSQI component scores are ordinal and were not normally distributed, nonparametric Wilcoxon signed-rank tests were consistently applied for component-level analyses. Given the clinical heterogeneity of the cohort, analyses were performed both for the overall population and separately for each treatment indication (healthy aging, long COVID, and PTSD). In addition, a multivariable linear regression model was performed with post-treatment PSQI as the dependent variable and baseline PSQI, age, sex, and treatment indication entered simultaneously as independent predictors in order to account for baseline clinical differences between groups. A *p*-value < 0.05 was considered statistically significant.

As this was a retrospective cohort study, no *a priori* sample size calculation was performed. All eligible patients with complete pre- and post-treatment PSQI data were included. Sample adequacy was evaluated based on the precision of the estimated change in PSQI.

Only participants with complete PSQI data at both baseline and post-treatment were included; no imputation of missing data was performed.

Given that analyses were conducted across the seven PSQI subscales, Bonferroni correction was applied for multiple comparisons (corrected significance threshold *α* = 0.007).

### Potential sources of bias

Because this was a retrospective observational study, several potential sources of bias should be considered. Selection bias may arise from including only patients who completed the HBOT protocol and had complete PSQI questionnaires, as patients with incomplete questionnaires were excluded from the analysis. Additionally, treatment was delivered as part of routine clinical care and not within a randomized design. These factors were considered when interpreting the results.

### Ethical considerations

This study received approval from Shamir Medical Center’s IRB. As a retrospective analysis of de-identified data, informed consent was waived.

## Results

Between January 2017 and January 2025, a total of 395 patients completed a 60-session hyperbaric oxygen therapy (HBOT) protocol and filled out the Pittsburgh Sleep Quality Index (PSQI) questionnaire both at baseline (prior to the first session) and at the end of the treatment course.

The mean age at baseline of the cohort was 57.9 ± 14.6 years, and 31% (*n* = 125) were female. Of the total, 180 patients received HBOT for healthy aging, 92 for long COVID symptoms, and 123 for post-traumatic stress disorder (PTSD). Baseline demographic and sleep quality characteristics are summarized in Table 1.

The overall PSQI score at baseline for the entire cohort was 9.0 ± 4.0. When stratified by indication, baseline scores were 7.1 ± 3.3 for healthy aging, 8.4 ± 3.6 for long COVID, and 12.1 ± 3.5 for PTSD. Following treatment, the global PSQI score improved significantly in all groups: to 6.5 ± 3.0 in the healthy aging group, 7.7 ± 3.3 in the long COVID group, and 10.9 ± 3.6 in the PTSD group (all *p* < 0.001). In the full cohort, the mean change in PSQI score was 0.8 points (SD = 0.88), with a 95% confidence interval ranging from 0.71 to 0.89, indicating a small but precisely estimated average improvement in sleep quality. Effect sizes for the global PSQI score ranged from moderate to large across study populations (Cohen’s d = 0.37–0.91), indicating a clinically meaningful treatment effect.

Significant improvements were also observed across most individual PSQI components (Figure 1). Following treatment, sleep disturbances improved significantly (Z = −13.29, *p* < 0.001), as did subjective sleep quality (Z = −7.8, *p* < 0.001), sleep latency (Z = −5.37, *p* < 0.001), and daytime dysfunction (Z = −9.12, *p* < 0.001). Although the mean sleep duration score remained numerically unchanged (1.6 vs. 1.6), the Wilcoxon signed-rank test indicated a modest shift in the paired distribution of scores (Z = −2.21, *p* = 0.027). No significant changes were observed in sleep efficiency (Z = −0.39, *p* = 0.73), Medication use, as reported in the questionnaire, did not change significantly (Z = −1.24, *p* = 0.24). After Bonferroni correction for multiple comparisons, improvements in sleep disturbances, sleep latency, and subjective sleep quality remained statistically significant across groups, whereas findings with *p*-values close to 0.05 should be interpreted cautiously.

To address concerns regarding regression to the mean and baseline severity differences between diagnostic groups, we performed a multivariable linear regression with post-treatment PSQI as the dependent variable and baseline PSQI, age, sex, and treatment indication as predictors. Baseline PSQI emerged as the strongest independent predictor of post-treatment sleep quality (B = 0.492, *p* < 0.001). Neither age, sex, nor treatment indication independently predicted post-treatment PSQI after adjustment. To further examine the impact of baseline sleep severity, participants with normal sleep at baseline (PSQI ≤ 5) were excluded from this subgroup analysis (*n* = 63) and the effect of HBOT on disturbed sleep (PSQI > 5) was evaluated. Among participants with elevated baseline PSQI scores (*n* = 332), significant improvements were observed for overall score, as well as across most sleep parameters as assessed using paired t-test for total PSQI and Wilcoxon for sleep parameters. Overall score decreased from 10.76 ± 3.46 to 8.82 ± 3.67 (*p* < 0.001). Sleep duration improved significantly (Z = −3.000, *p* = 0.003). Sleep disturbances showed a large and highly significant improvement (Z = −13.081, *p* < 0.001). Daytime dysfunction improved significantly (Z = −4.466, *p* < 0.001), as did subjective sleep quality (Z = −7.849, *p* < 0.001). Sleep latency also improved significantly (Z = −6.414, *p* < 0.001), reflecting reduced time to fall asleep. No statistically significant changes were observed in sleep efficiency (Z = −1.291, *p* = 0.197) or use of sleep medications (Z = −1.321, *p* = 0.186).

These findings suggest that among individuals with disturbed sleep at baseline, HBOT led to clinically and statistically significant improvements in multiple aspects of sleep, particularly sleep disturbances, latency, and subjective sleep quality.

## Discussion

The current study evaluated the effects of hyperbaric oxygen therapy (HBOT) on sleep parameters, as assessed through a validated sleep assessment questionnaire. Findings demonstrated a significant and robust improvement in sleep quality across diverse clinical populations, suggesting that HBOT was associated with improved sleep quality and supporting the need for prospective controlled trials to validate efficacy, identify patients most likely to benefit, and elucidate underlying mechanisms.” One possible explanation for the observed improvements in sleep is the amelioration of the primary condition for which HBOT was administered. For example, sleep disturbances are a core symptom of PTSD. As HBOT has been shown to reduce the overall symptom burden in PTSD (16, 17), this may secondarily lead to reductions in nightmares, improved sleep initiation and maintenance, and enhanced subjective sleep quality.

A second, broader potential explanation is that HBOT improves sleep through generalized enhancement of brain function, including modulation of autonomic balance (32), mitochondrial activity (15, 33, 34), inflammation (35), and neuroplasticity (15, 36). HBOT induces angiogenesis VEGF and HIF pathways, which may directly influence neuronal perfusion and energy metabolism (15, 37, 38), thereby promoting overall neuronal health and sleep quality. Previous studies have linked disrupted mitochondrial function to sleep disorders (39), supporting the notion that enhanced mitochondrial biogenesis may play a role in the observed improvements. Additionally, HBOT’s anti-inflammatory effects, both systemic and neuroinflammatory, are relevant given the established links between inflammation, circadian disruption, and impaired sleep. Enhanced neuroplasticity and upregulation of neurotrophic factors such as brain-derived neurotrophic factor (BDNF) following HBOT may also contribute to improvements in sleep architecture and quality (40, 41).

A third potential mechanism involves direct effects of HBOT on sleep-regulatory pathways, such as adenosine accumulation and melatonin signaling. Adenosine functions as a key neuromodulator promoting sleep (42), and increased tissue oxygen availability may influence adenosine metabolism and receptor signaling (1). Preliminary findings also suggest that HBOT affects melatonin receptor activity, potentially enhancing circadian alignment and sleep onset (43).

An important factor to consider in interpreting these results is treatment dosage. All patients in this study completed 60 daily HBOT sessions. Evidence from other populations indicates that a sufficient cumulative oxygen dose is critical to induce durable neuroplastic changes and long-term clinical effects (44, 45). For example, prior studies in individuals with long COVID and in children with cerebral palsy using only 10 HBOT sessions did not demonstrate improvements in sleep parameters. However, in the CP study, 20 sessions did result in measurable benefits (25). Same with relation to patients who are suffering from long COVID, unlike 40 HBOT sessions found to be effective (19) 10 sessions protocol was not (46). This suggests a dose–response relationship between cumulative oxygen exposure (measured in atmosphere-minutes) and symptomatic improvement, including for sleep.

The study’s limitations are primarily related to its retrospective design and the absence of a control group, which preclude causal inference regarding the effects of HBOT on sleep outcomes. Consequently, the observed improvements in PSQI scores should be interpreted cautiously and considered as associations rather than definitive evidence of therapeutic efficacy. Changes in patient-reported sleep quality may reflect regression to the mean, natural fluctuations in sleep symptoms over time, expectancy or placebo effects, or other non-specific aspects of participation in a structured treatment program involving daily clinical interaction and monitoring. HBOT in this cohort was delivered as part of routine clinical care rather than as an isolated experimental intervention. Accordingly, the observed changes in sleep quality may reflect both direct physiological effects of HBOT and indirect effects related to participation in a structured clinical environment. In addition, sleep outcomes in this study were assessed exclusively using the self-reported PSQI. Although the PSQI is a well-validated and widely used instrument for evaluating perceived sleep quality, it reflects subjective sleep experience rather than objective sleep physiology. Subjective assessments of sleep quality may diverge from objectively measured sleep parameters such as sleep architecture, sleep efficiency, or fragmentation measured using instrumental methods including polysomnography or sleep polygraphy. Several studies have demonstrated that patient-reported sleep disturbances do not always correspond to objective sleep metrics in the same individuals, highlighting the complex relationship between perceived and physiological sleep quality (47, 48). This discrepancy may arise from multiple factors, including cognitive and emotional influences on sleep perception, underlying neuropsychiatric conditions, and thus, recent studies have emphasized that subjective and objective sleep measures capture partially distinct aspects of sleep health and should ideally be interpreted as complementary rather than interchangeable assessments. In addition, given the psychological and contextual influence on subjective sleep assessments, patient-reported sleep quality may be affected by mood, stress levels, expectations regarding treatment efficacy, and the broader therapeutic environment in which treatment occurs. In the context of an uncontrolled retrospective cohort, improvements in PSQI scores may therefore reflect not only physiological changes in sleep but also expectancy effects or placebo-related influences associated with participation in an intensive treatment program.

Accordingly, reliance solely on questionnaire-based evaluation introduces the possibility of measurement bias and limits the ability to determine whether the observed improvements reflect changes in sleep physiology, sleep perception, or both. Future prospective, controlled studies investigating the effects of HBOT on sleep should incorporate objective sleep assessments such as actigraphy, sleep polygraphy, or polysomnography alongside validated questionnaires in order to provide a more comprehensive characterization of sleep outcomes.

Potential confounding by baseline comorbidities and pharmacotherapy should also be considered. The study population demonstrated variability in medical conditions and medication use, including antidepressants, benzodiazepines, and cardiovascular medications, which may independently influence sleep quality. Although the multivariable regression analysis adjusted for major demographic and clinical variables including baseline PSQI and treatment indication, residual confounding cannot be fully excluded in a retrospective observational design.

An additional limitation is the lack of detailed longitudinal pharmacotherapy data. While overall sleep medication use was captured via the PSQI questionnaire, information regarding specific medications, dosages, and changes in pharmacological treatment during the HBOT course was not available; therefore, potential medication-related effects on sleep outcomes cannot be fully excluded.

## Conclusion

This retrospective analysis suggests that hyperbaric oxygen therapy was associated with improvements in patient-reported sleep quality across several clinical populations. However, due to the observational design and the absence of a control group, causal conclusions cannot be drawn. These findings support the need for prospective randomized controlled trials incorporating objective sleep assessments to better characterize the potential role of HBOT in modulating sleep and to identify patient populations that may benefit the most.

## Data Availability

The datasets generated and/or analyzed during the current study are available from the corresponding author on reasonable request. Requests to access these datasets should be directed to KD-B Kerendoenyas@gmail.com.
